# Supplementary material for: 3D-Printed Pectin/Carboxymethyl Cellulose/ZnO Bio-Inks: Comparative Analysis with the Solution Casting Method
Source: Polymers (Basel). 2022 Nov 3;14(21):4711. doi: 10.3390/polym14214711 (PMC9657909; doi:10.3390/polym14214711)
Supplement: Supplementary file 1 [file polymers-14-04711-s001.zip › polymers-1981524-supplementary.pdf]

polymers

MDPI

Supplementary Materials

Thermal Analysis of Pec/CMC-based films

The thermal analysis of the neat and nanocomposite Pec/CMC-based films was carried out within a range 35 °C–150 °C to determine their moisture content. It has been reported that the temperature dependent weight loss in the biopolymeric films below 150 °C is due to the evaporation of moisture from the films, hence determines the moisture content of the films [1]. It can be seen from Figure S1, that the weight loss due to moisture evaporation from the solution cast films was more than that from the 3D-printed films. The weight of moisture lost from the films at 150 °C was found to be 14% and 13% for Pec/CMC-SC and Pec/CMC/ZnO-SC, respectively. On the other hand, 10% and 9% moisture weight were lost for Pec/CMC-3D and Pec/CMC/ZnO-3D, respectively. This clearly indicates a higher moisture content in solution cast films than 3D printed films.

Further, it can be seen that both types of ZnONPs incorporated films have a lower moisture content than the neat films. This indicates that the -OH groups in the polymer structure, which are responsible for interacting with water molecules and binding with them, may not be available to bind moisture when ZnONPs are incorporated. This confirms the presence of H-bonding between the nanoparticles and hydroxyl groups in polymer structure, thus occupying the free -OH groups.

The moisture content in the films play a major role in affecting their physicochemical properties such as mechanical properties. Since bound moisture acts as a plasticizing agent [2], the TS of the films with more water content tend to be lower compared to those with less water content.

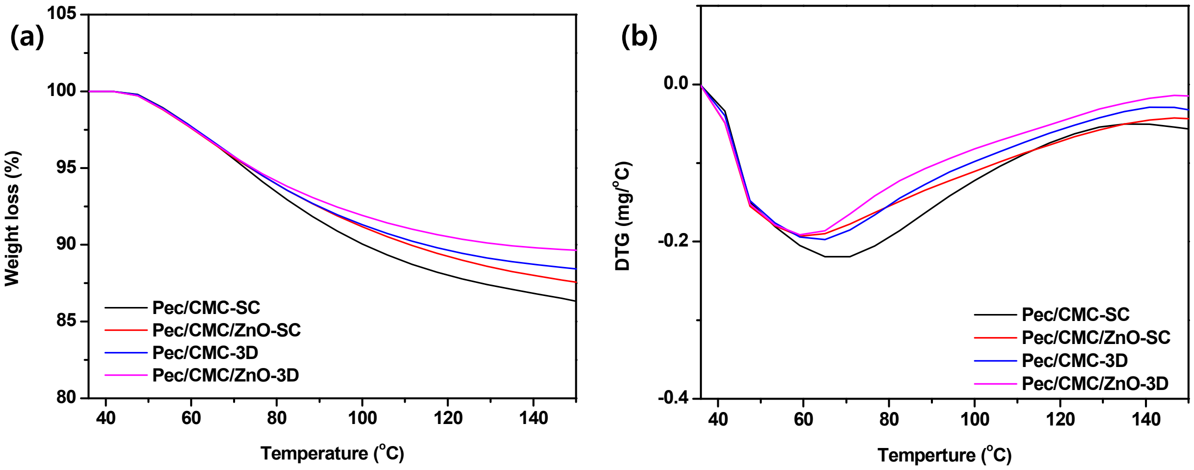

Figure S1. (a) TGA and (b) DTG curves for Pec/CMC-based solution cast and 3D-printed films.

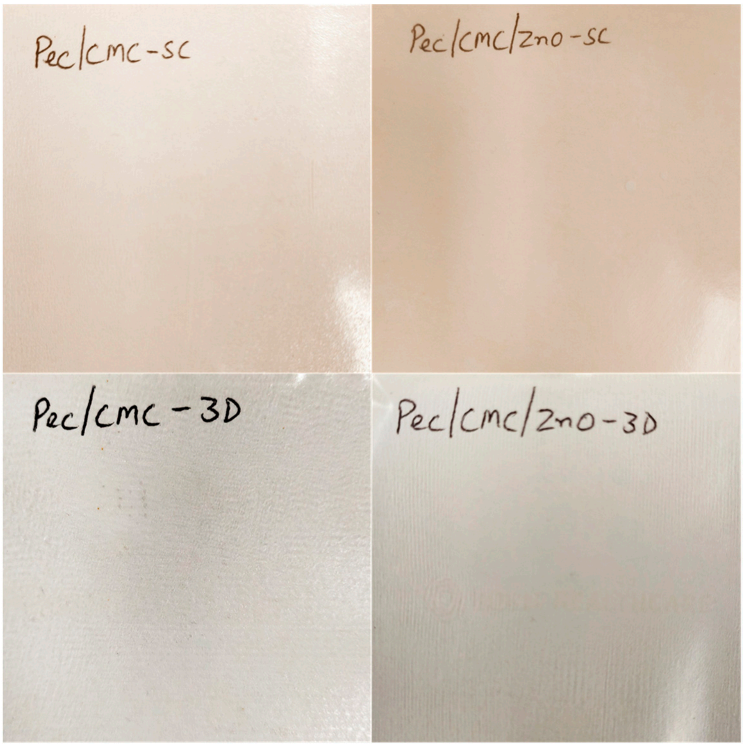

Figure S2. Digital images of Pec/CMC-based solution cast and 3D-printed films.

References

1. Priyadarshi, R.; Sauraj; Kumar, B.; Negi, Y.S. Chitosan Film Incorporated with Citric Acid and Glycerol as an Active Packaging Material for Extension of Green Chilli Shelf Life. *Carbohydr. Polym.* **2018**, *195*, 329–338, doi:10.1016/j.carbpol.2018.04.089.
2. Vieira, M.G.A.; Da Silva, M.A.; Dos Santos, L.O.; Beppu, M.M. Natural-Based Plasticizers and Biopolymer Films: A Review. *Eur. Polym. J.* **2011**, *47*, 254–263, doi:10.1016/J.EURPOLYMJ.2010.12.011.
